# Supplementary material for: Crawling and Gliding: A Computational Model for Shape-Driven Cell Migration
Source: PLoS Comput Biol. 2015 Oct 21;11(10):e1004280. doi: 10.1371/journal.pcbi.1004280 (PMC4619082; doi:10.1371/journal.pcbi.1004280)
Supplement: S1 Code — (ZIP) [file pcbi.1004280.s012.zip › release/tst/doc/html/functions_m.html]

Tissue Simulation Toolkit: Class Members


Tissue Simulation Toolkit
 0.1.4.1


- Main Page
- Namespaces
- Classes
- Files

- Class List
- Class Hierarchy
- Class Members

- All
- Functions
- Variables
- Related Functions

- a
- b
- c
- d
- e
- f
- g
- i
- j
- l
- m
- n
- o
- p
- q
- r
- s
- t
- v
- w
- x
- y
- z
- ~

Here is a list of all class members with links to the classes they belong to:

### - m -

- MapColour()
  : PDE
- Mass()
  : CellularPotts
- Max()
  : PDE
- maxsigma
  : Cell
- MaxSigma()
  : Cell
- maxtau
  : Cell
- mcs
  : Parameter
- MeanCellArea()
  : CellularPotts
- MeasureCellSize()
  : Cell
- MeasureChemConcentrations()
  : Dish
- Menu()
  : Info
- Min()
  : PDE
- Morphometry
  : CellularPotts
- Mother()
  : Cell
- mother
  : Cell


---

Generated on Thu Aug 14 2014 22:04:02 for Tissue Simulation Toolkit by  

 1.8.6
